# Mechanisms of complex regional pain syndrome

**DOI:** 10.3389/fpain.2024.1385889

**Published:** 2024-05-17

**Authors:** Jagan Devarajan, Shayla Mena, Jianguo Cheng

**Affiliations:** ^1^ Department of Pain Management, Neurological Institute, Cleveland Clinic, Cleveland, OH, United States; ^2^ Department of Pain Management and Neurosciences, Neurological Institute, Cleveland Clinic, Cleveland, OH, United States

**Keywords:** complex regional pain syndrome, neuroimmune interactions, neuroinflammation, autonomic dysfunction, pathophysiology, autoimmune disorder, genetic predisposition

## Abstract

Complex Regional Pain Syndrome (CRPS) is a chronic pain disorder characterized by a diverse array of symptoms, including pain that is disproportionate to the initial triggering event, accompanied by autonomic, sensory, motor, and sudomotor disturbances. The primary pathology of both types of CRPS (Type I, also known as reflex sympathetic dystrophy, RSD; Type II, also known as causalgia) is featured by allodynia, edema, changes in skin color and temperature, and dystrophy, predominantly affecting extremities. Recent studies started to unravel the complex pathogenic mechanisms of CRPS, particularly from an autoimmune and neuroimmune interaction perspective. CRPS is now recognized as a systemic disease that stems from a complex interplay of inflammatory, immunologic, neurogenic, genetic, and psychologic factors. The relative contributions of these factors may vary among patients and even within a single patient over time. Key mechanisms underlying clinical manifestations include peripheral and central sensitization, sympathetic dysregulation, and alterations in somatosensory processing. Enhanced understanding of the mechanisms of CRPS is crucial for the development of effective therapeutic interventions. While our mechanistic understanding of CRPS remains incomplete, this article updates recent research advancements and sheds light on the etiology, pathogenesis, and molecular underpinnings of CRPS.

## Introduction

Complex Regional Pain Syndrome (CRPS) is a debilitating chronic pain condition that often perplexes both patients and healthcare professionals with its complex array of symptoms and poorly understood mechanisms. Characterized by disproportionate pain relative to the initial injury or trauma, along with a myriad of autonomic, sensory, motor, and sudomotor disturbances, CRPS presents significant challenges in diagnosis and management. The disorder manifests in two main types: CRPS Type I, occurring without a discernible nerve injury, and CRPS Type II, following a distinct nerve injury. Recent studies have begun to unravel its multifaceted nature, highlighting autoimmune mechanisms and neuroimmune interactions. We have recently reviewed the role of neuroinflammation in CRPS (1). Here we aim to provide a comprehensive review of the enigmatic nature of CRPS, setting the stage for further exploration into its complexities and advancements in understanding and treating this debilitating condition.

The identification and evolution of CRPS represent a significant journey of understanding and addressing a complex and often misunderstood condition. Over time, advancements in research, diagnostic techniques, and multidisciplinary approaches have led to improved recognition and management of CRPS. Through collaborative efforts among clinicians, researchers, and patients, there has been a growing understanding of the underlying mechanisms, contributing factors, and treatment modalities for CRPS. However, ongoing research and education are essential to further enhance our understanding and refine therapeutic strategies for individuals affected by this debilitating condition.

The legacy of identifying CRPS exemplifies a milestone in medical discovery, showcasing the intricate nature of diagnosing and understanding complex diseases. Ambroise Paré, often called the “Father of Modern Surgery” was the first to describe a disorder seemingly like CRPS in the sixteenth century (2). However, it wasn't until the American Civil War that physician Sila Wier Mitchell described several cases of what is now believed to be CRPS. His monograph, “Gunshot Wounds and Other Injuries,” would go on to become the benchmark for diagnosing nerve damage until World War I (3). Mitchell later coined the term “causalgia” for this disease in 1872 (4). Following that in 1900, German surgeon Paul Sudeck presented a paper at the 29th Congress of the German Society of Surgery (“Acute inflammatory bone atrophy”) describing a particular type of bone atrophy (5). The following year, his student called this pathologic phenomenon “Sudeck's atrophy” (5, 6). Over the following decades, various surgeons and physicians recognized the role of sympathetic activity and sympathectomy in several chronic pain syndromes (7–9).

John Bonica published “The Management of Pain”, and proposed staging for RSD in 1953 (10), and later started the first scientific society devoted exclusively to the study of pain in 1973 (The International Association for the Study of Pain, IASP) (5). Bonica renamed the disease as “Complex Regional Pain Syndrome” during the 1993 IASP Orlando Conference to emphasize that the predominant aspect of the disease was the localization of pain in a particular anatomic region (11). The Orlando Conference Criteria yielded a high sensitivity (close to 90%), but a low specificity (less than 50%) resulting in misdiagnosing diseases as CRPS (5). It wasn't until the Budapest Conference in 2003 that what is now known as the most accepted diagnostic criteria for CRPS was developed (Appendix 1). The Budapest Criteria enhanced the specificity of earlier criteria (99% sensitivity and 68% specificity), although some literature suggests it may not be generalizable to all populations, particularly post-stroke CRPS (12, 13). Other diagnostic criteria that have been proposed for CRPS include those by Dutch surgeon Peter Veldman and British surgeon Roger Michael Atkins (14, 15).

## Clinical manifestations, progression, and diagnosis

CRPS is the current diagnostic label for the constellation of signs and symptoms that has historically been referred to as RSD, reflex neurovascular dystrophy, causalgia, Sudeck's atrophy, algodystrophy, algoneurodystrophy, and shoulder-hand syndrome (16). It is a chronic pain disorder that is distinguished by its autonomic as well as structural features (17). CRPS typically affects an extremity after a traumatic injury, with a propensity for spreading to alternate anatomical sites (18), including the contralateral side, as recognized by the National Institute of Neurological Disorders and Stroke (https://www.ninds.nih.gov/health-information/disorders/complex-regional-pain-syndrome). Knowledge of the spreading pattern of CRPS may lead to hypotheses about underlying mechanisms (19). Predominantly, CRPS occurrences manifest in the extremities, yet instances have been documented in the orofacial and neck regions (20–22). CRPS can even arise spontaneously without an instigating event or known cause. Interestingly, CRPS has been described in patients who have suffered strokes, indicating the complexity of the pathophysiology involved in the disease process (23).

The manifestations of Type I and Type II CRPS are similar despite the diagnostic distinction between the two types. In addition to the pain characteristics (e.g., burning pain, hyperalgesia, and allodynia), local edema, skin discoloration, altered sweating, temperature abnormalities in the affected region, trophic changes (e.g., change in skin, hair, or nail growth), and altered motor function (e.g., loss of strength, decreased active range of motion, and tremor) can all occur. The goals and strategies for the treatment of CRPS remain the same irrespective of the types. The objectives of the intervention are not only achieving amelioration of pain and discomfort but also restoration of functionality and mitigation of disability. Treatment of CRPS does not differ between the two types. However, treatment of CRPS Type II, in addition, may include treating the underlying nerve injury whenever possible (24). Early diagnosis and treatment typically lead to better outcomes. Studies also indicate that pain and motor dysfunction are the most dominant long-term features of CRPS, persisting for 51%–89% of patients greater than or equal to 12 months from symptom onset (25).

## Epidemiology, genetic and environmental risk factors

The variability in the prevalence of CRPS can be attributed to inconsistencies in the employed diagnostic criteria. The differing sensitivity and specificity of these criteria influence the reported incidence. For instance, the Orlando criteria demonstrate a high sensitivity with low specificity, while the Budapest criteria maintain a high sensitivity while significantly enhancing specificity (26). This disparity in diagnostic criteria contributes to the observed variation in reported incidence rates, particularly in studies published before the compilation of the Budapest criteria. It is worth highlighting that the mentioned data specifically pertains to CRPS Type I, excluding CRPS Type II from consideration in many studies (27).

CRPS is almost three or four times more common in women than in men, and peaks in onset between the ages of 50 and 70 years (28). Given the changes in diagnostic criteria and the evolving understanding of the disease process, studies vary on the actual incidence of CRPS. The overall incidence rates of CRPS can range anywhere from approximately 5 to 29 per 100,000 people each year, with the highest incidence typically occurring in females between 61 and 70 years of age (13, 29, 30). Based on the epidemiological studies available, it appears to be more common in patients of North European ancestry, although it does appear to occur frequently in those of South Korean ancestry as well (29, 30). In the United States, it is estimated that there are at least 50,000 cases of CRPS type I each year (31). Similarly, with the variation in CRPS diagnostic criteria over the years the incidence among patients with fractures is anywhere from 0.05% to 0.2% in older studies and 3% to 7% in more recent studies (32). The upper extremity is affected more frequently than the lower extremity (29). Although CRPS can develop after any injury, the most common initiating events are fractures, surgery, crush injuries, and sprains (33).

Genetic factors have been increasingly shown to play a role in the development of CRPS. It's known to occur in multiple family members. When comparing patients with sporadic CRPS to families where two or more patients are affected, familial CRPS patients had a younger age at onset and more often had multiple affected extremities and dystonia (34). A recent study demonstrated that a single nucleotide polymorphism in four genes, ANO10, P2RX7, PRKAG1, and SLC12A9, is associated with developing CRPS Type I with males typically expressing these rare alleles (35). Other genetic studies have demonstrated associations between CRPS and several major histocompatibility complex alleles, including human leukocyte antigen (HLA)-DR6, HLA-DR13, HLA-DR2, HLA-DQ1, HLA-B62, and HLA-DQ8 (36). Genome-wide expression profiling of the whole blood has shown that HLA-A29.1, matrix metallopeptidase 9 (MMP9), alanyl aminopeptidase (AAP), histidine decarboxylase (HDC), granulocyte colony-stimulating factor 3 receptor (G-CSF3-R), and signal transducer and activator of transcription 3 genes (STAT-3) were highly expressed when compared to unaffected controls (37). Further genetic and transcriptomic studies hold the promise to predict patient predispositions to CRPS, unravel the molecular mechanisms of CRPS pathogenesis, and discover novel therapeutic targets for CRPS (38).

Psychosocial and environmental factors may predispose patients to developing CRPS. Patients who experience more stressful life events have higher chances of developing CRPS (39). In children, CRPS is more likely to be associated with a higher number of stressful life events when compared to chronic primary headaches and functional abdominal pain (40). Individuals with post-traumatic stress disorder (PTSD) exhibit a markedly higher occurrence of CRPS in comparison to control groups (41). Moreover, individuals diagnosed with CRPS who exhibit elevated levels of anxiety, perception of disability, fear of movement (kinesiophobia), and apprehension towards pain have been discovered to experience a deteriorating trajectory of their condition (42). This observation can potentially be attributed to the heightened activity of catecholamines, thus exacerbating the process of nociceptive sensitization. Catastrophizing, in a similar vein, has also been associated with heightened pain intensity among individuals with CRPS. However, in contrast to the above studies, a prospective and comprehensive study conducted across multiple centers did not establish any correlation between the existence of psychological elements (such as agoraphobia) and CRPS (43). Thus, while psychological or personality traits cannot be considered independent risk factors for CRPS, preexisting anxiety and mood disorders are risk factors for CRPS Type I after upper or lower extremity fractures (44, 45). Overall, Axis I disorders, and particularly major depression are present in up to 49% of CRPS patients (46). However, there is no evidence that comorbid psychiatric disorders are more common in patients with CRPS when compared to other patients who have chronic pain (46).

In a systematic review, risk factors for CRPS Type I from studies with higher quality evidence were: being female (particularly postmenopausal), fracturing the distal radius, suffering an ankle dislocation or intra-articular fracture, and reports of higher than usual levels of pain in the early phases after trauma (44). For poststroke CRPS, a meta-analysis identified being female, left hemiparesis, shoulder subluxation, spasticity, a lower Brunnstrom stage of the distal upper limb, and inferior Barthel index as risk factors (47). However, contradicting findings from a study suggest that CRPS is not more prevalent among individuals with radius fractures compared to the general population (48). This highlights the complexity of the relationship between injury type, severity, and the subsequent development of CRPS.

## Pathophysiology

**Figure 1:**
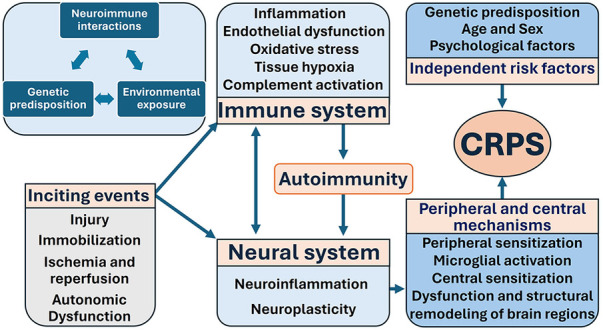
Mechanisms of CRPS highlight the three key components: neuroimmune interactions, genetic predisposition, and environmental exposure. CRPS is the result of complex interplay between inciting events, the immune system, the neural system, genetic predisposition, and other independent risk factors.

CRPS symptomatology varies, and the diversity of the symptoms cannot be adequately explained by a single pathophysiological mechanism. The etiology and pathogenesis of CRPS may exhibit inter-individual heterogeneity and even intra-individual variability over time (44, 49, 50). The most common inciting events are surgery, nerve compression, fractures, tissue trauma, ischemia, and sprains (27, 51).4 Inflammation (52), oxidative stress (53), and neuronal mechanisms have been postulated as pivotal factors in the pathogenesis of CRPS (50, 54). Current evidence suggests that the development of CRPS involves multiple mechanisms originating from a complex interplay between the immune system, the neural systems (including the peripheral nervous system (PNS), central nervous system (CNS), and autonomic nervous system), and genetic predisposition (55) (Figure 1).

CRPS is a form of “nociplastic pain”, a concept newly introduced by the IASP pain taxonomy (56, 57). This is supported by evidence of changes in cerebral connectivity affecting nociception, even without any discernable tissue damage or alterations in the somatosensory system (58). As aforementioned, it is also likely that psychological, environmental, and genetic factors play a role in influencing CRPS symptoms. The hallmark of the initial phase of CRPS is an exaggerated inflammatory response to the initial trauma. CRPS manifests with all the cardinal signs of inflammation, including pain, edema, erythema, increased temperature, and impaired function (49, 59). Pro-inflammatory cytokines such as interleukin (IL)-1β, IL-2, IL-6, and tumor necrosis factor-α (TNF-α), as well as neuropeptides like calcitonin gene-related peptide (CGRP), bradykinin, and substance P, contribute to the intense inflammatory response following trauma or injury, ultimately instigating the development of CRPS (60, 61).

### Injury

Various injury mechanisms leading to tissue damage, subsequent inflammation, and even the distinct recovery process contribute to the risk of developing CRPS. There is an intricate correlation between the onset of CRPS and injury, its location, and its mechanism. Interestingly, the severity of the initial injury does not correlate with the likelihood of developing CRPS or the subsequent pain intensity (32, 62). Even seemingly minor injuries like intramuscular injections have been documented as evolving into CRPS.

Trauma and other injuries constitute 75% of total cases of CRPS. Fractures account for approximately 45% of reported triggering events, followed by sprains at around 18% and elective surgery at approximately 12% (49, 63). On the other hand, spontaneous onset, which manifests with a similar clinical presentation, is rare and occurs in less than 10% of cases (29). Trauma-induced tissue injury increases the risk of the development of CRPS by nearly threefold (odds ratio, 2.96; 95% confidence interval, 2.18–4.02; *p* < 0.05), independent of factors such as age, sex, and other associated risk factors. In addition, tissue injury exhibited a positive correlation with various other risk factors, including headache, osteoporosis, myofascial pain, anxiety, and preexisting neuropathy (64). After adjustment for confounding variables, it was noted that specific types of injuries, including open wounds of the upper limbs, sprains, and strains of the joints and adjacent muscles, superficial injuries, contusions with intact skin surfaces, as well as injuries to nerves and the spinal cord, were associated with an elevated risk of developing CRPS. While CRPS Type II is typically categorized by identifiable nerve injury, a notable proportion of trauma- and surgery-related CRPS cases are classified as Type I, despite demonstrable peripheral nerve fiber damage associated with injury. This caveat further precipitates the lack of studies available to investigate both CRPS Type I and Type II since the latter is infrequently diagnosed. However, pathological investigations of chronically affected CRPS limbs, including amputated tissue and skin biopsies, have provided unequivocal evidence of small nerve fiber (C and Aδ) degeneration. This reveals direct small-fiber nerve damage due to injuries (65). Furthermore, CRPS is suspected to arise from an imbalance between pro-inflammatory and anti-inflammatory cytokines. The longer this imbalance persists, the greater the likelihood of developing CRPS. Among CRPS patients, elevated skin TNF-α levels were observed and endured for months following limb trauma (66). In contrast, in patients with burns, the imbalance peaks but does not endure for an extended period, resulting in a lower risk of CRPS.

### Immobilization

Immobilization is suggested as a potential risk factor for the onset of CRPS (67). Several hallmark features of CRPS such as altered temperature, mechano-sensitivity, and thermos-sensitivity can be transiently induced in healthy limbs solely through immobilization. Healthy volunteers exhibited mild CRPS-like symptoms (excluding pain) when they were subjected to four weeks of limb immobilization even without trauma (68). In a rat model of tibial fracture, immobilization led to increased levels of substance P and IL-1β. Inflammasome multiprotein complexes containing caspase-1 and NACHT leucine-rich-repeat protein 1 (NALP1), activated by NK1 receptors, were expressed in keratinocytes, indicating the involvement of innate immunity in the development of CRPS (69). Furthermore, topical capsaicin application, known to induce neurogenic inflammation, resulted in mechano-sensitivity, thermal sensitivity, and perceptual disturbances only when followed by 24 h of limb immobilization. The above signs did not appear if the limb was mobilized sooner, and these symptoms swiftly resolved upon limb remobilization (49). These experimental findings strongly support the notion that immobilization serves as a significant risk factor for CRPS development. In a prospective multicenter cohort study where 88.8% of fracture patients underwent conservative treatment with plaster casts or taping, the incidence of CRPS after fracture was 7% (32). In contrast, when the fracture was managed by early surgery, followed by active mobilization, only 4.5% of patients developed CRPS (64). Postoperatively, patients tend to experience an improved range of motion due to reduced pain and increased stability. Consequently, immobilization could be considered another factor influencing the relationship between fracture and the development of CRPS.

### Ischemia-reperfusion

CRPS symptoms can be detected in the context of ischemic injury arising from ischemia-reperfusion processes in myocardial infarction (70). This phenomenon has been extensively described, and it has been observed that as an inflammatory response develops, arteriole spasms develop. This is caused by a change in the expression of *α*-adrenoceptors on arterial smooth muscle cells, as well as imbalances between nitric oxide (NO) and endothelin-1 (ET-1). Consequently, capillaries can become occluded due to endothelial damage, leading to a loss of function in small nerve fibers because of ischemia within the endoneurium (71). The normal response following ischemia-reperfusion processes in arterioles, capillaries, and venules becomes decreased due to interactions and negative feedback loops, all of which contribute to the enhancement of local ischemia (72). Resulting endothelial dysfunction could develop leading to abnormal vascular tone in CRPS. Endothelium releases both endothelium-derived vasodilators, such as NO, bradykinin, prostacyclin, and endothelium-derived hyperpolarizing factor (EDHF), and vasoconstrictors such as ET-1 and angiotensin II (ATII) (73). Hence endothelial dysfunction because of inflammation could cause imbalances in vascular tonicity and ischemia (72). Severe damage to the deep tissues can lead to the development of edema and local compartment syndrome, thereby further impairing tissue oxygenation and exacerbating the damage caused by ischemia and reperfusion, along with all its consequences (74).

### Autonomic dysfunction

Two stages of CRPS have been identified: the initial warm phase followed by the chronic and cold phase (75). During the initial acute or “warm” phase, affected tissues show classic signs of inflammation in the affected limb—color, dolor, rubor, and tumor. The symptoms typically manifest distally to the site of injury, resembling a glove or stocking. Patients report continuous, deep pain worsened by movement or changes in temperature (15).

The subsequent chronic or “cold” phase typically emerges approximately 6 months later, following a decrease in inflammation. The nature of the pain changes. Patients would manifest with more persistent pain at rest, which could be challenging to manage. Some individuals may experience muscle spasms. Skin, subcutaneous tissue, and muscle atrophy may occur, along with localized osteoporosis in the underlying bones. Nail and hair growth may be altered, either increasing or decreasing in conjunction with changes in quality (76).

Autonomic manifestations correspond with the above two phases. Sudomotor symptoms include excessive followed by reduced sweating and alterations in skin color, particularly the limb turning red in the beginning, which turns into pale, purple, and cyanotic in later stages (77). Motor impairments commonly accompany Complex Regional Pain Syndrome (CRPS) phases as well: during the initial phase, movement is limited due to swelling and fear of exacerbating pain (kinesiophobia), while fibrosis in the chronic stage further restricts movement (78).

One of the characteristic features of CRPS is cold clammy and cyanotic extremities, which occurs during the second phase and may be mediated by excessive sympathetic nervous system activation. Animal studies have shown increased adrenergic receptor expression on nociceptive fibers following nerve trauma. This promotes heightened sympathetic activation and sustains pain induced by sympathetic activity resulting in causalgia (79). In addition, sympatho-afferent coupling can be explained by increased expression of adrenergic receptors on nociceptive fibers following injury. Hence sympathetic activation induces nociceptive fibers causing increased pain in most patients with CRPS (33, 80). Elevated sympathetic nervous system activity may amplify spontaneous pain by 22%. Additionally, there is a 42% increase in the spatial extent of dynamic hyperalgesia and a 27% increase in punctate hyperalgesia with heightened sympathetic nervous system function (81).

CRPS may progress from a warm acute phase to a cold phase as time elapses, a phenomenon that can be attributed to the imbalance between levels of circulating catecholamines and the peripheral adrenergic receptors (33). In the acute phase, there is a notable decrease in the levels of circulating plasma norepinephrine in the CRPS-affected extremities when compared to the unaffected ones (82). This consequently leads to compensatory upregulation of peripheral adrenergic receptors, resulting in heightened sensitivity to circulating catecholamines (83, 84). Following the subsiding of the acute phase and the restoration of catecholamine levels, excessive vasoconstriction and sweating occur, thereby manifesting as the characteristic cold and blue extremity observed during the chronic phase. Additionally, the administration of phenylephrine through intradermal injection elicits the sensation of pain and allodynia in limbs affected by CRPS (85). Successful pain reduction (more than 50%) was observed following sympathetic plexus block in 155 patients with CRPS (155 of 255, 61%). Most patients (132 of 155, 85%) experienced more than 50% pain relief for 1 to 4 weeks or longer. The degree and duration of pain relief were not associated with pre-procedure temperature parameters of the limbs with an estimated odds ratio of 1.03 (97.5% CI, 0.95–1.11) or 1.01 (97.5% CI, 0.96–1.06) for one-degree decrease (*P* = 0.459 or 0.809) (86). We also investigated whether outcomes of sympathetic nerve blocks can predict responders of spinal cord stimulation and found that there was no difference in the success rate of spinal cord stimulation trials between patients with or without more than 50% pain relief after sympathetic blocks (35 of 40, 88% vs. 26 of 29, 90%, *P* > 0.990) (86). In a multicenter study, we further investigated the demographic and clinical factors of sympathetic blocks as a predictor for response to ketamine infusion, a treatment frequently used in clinical practice (87), in patients with CRPS (80, 87). Factors associated with a positive response to ketamine in univariable analysis were the presence of sympathetically mediated pain (SMP) [61.0% success rate vs. 26.7% in those with sympathetically independent pain (SIP); *P* = .009] and post-block temperature increase (5.66 ± 4.20 in ketamine responders vs. 3.68 ± 3.85 in non-responders; *P* = .043). No psychiatric factor was associated with ketamine response. In multivariable analysis, SMP (OR 6.54 [95% CI: 1.83, 23.44) and obesity (OR 8.75 [95% 1.45, 52.73) were associated with a positive ketamine infusion outcome. From these studies, we conclude that sympathetic blocks may be therapeutic in patients with complex regional pain syndrome regardless of pre-procedure limb temperatures. The effects of sympathetic blocks do not predict the success of spinal cord stimulation but may predict response to ketamine infusion in CRPS patients.

### Inflammation

The afore-described inciting events may all lead to tissue inflammation. As a cardinal feature of CRPS, inflammation manifests as heightened local, systemic, and neural inflammation. Neuroinflammation can occur in both the CNS and PNS (15, 88). Patients with CRPS manifest with an elevated level of proinflammatory cytokines, including IL-1β, IL-6, and TNF-α, and a decrease in anti-inflammatory factors, including IL-10 cytokines in local blister fluid, circulating plasma, and cerebrospinal fluid (66, 89). In addition, the administration of corticosteroids and TNF-α antibodies significantly ameliorated the symptoms of CRPS (90). Immune cells activated by inflammation generate reactive oxygen species (ROS), which subsequently results in an imbalance in redox status and oxidative injury (91), contributing to maintaining inflammation and leading to a vicious circle culminating in excessive oxidative stress. There is substantial evidence demonstrating that the occurrence of neurogenic inflammation and stimulation of the immune system contributes significantly to the mechanisms underlying CRPS (61). This is supported by previous studies that have observed an increased systemic level of CGRP and plasma bradykinin in patients with CRPS compared to healthy individuals (61).

In a rat model of CRPS with a fractured tibia, chronic unilateral hindlimb warmth, and edema facilitated protein extravasation, allodynia, unweighting, and periarticular osteoporosis– a combination of nociceptive, vascular, and bone changes closely resembling CRPS (92, 93). Subsequent studies revealed elevated levels of IL-1β and other cytokines in the hind paw skin of the fractured limb (73). Similar increases in inflammatory cytokine levels were found in the affected limb of CRPS patients, consistent with the animal model (88). Furthermore, continuous administration of the IL-1 receptor antagonist (IL-1ra) anakinra reduced fracture-induced nociceptive sensitization in the rat fracture model. In situ hybridization and immunostaining indicated that epidermal keratinocytes were the primary source of IL-1β (94). Despite these findings, the mechanisms underlying the post-traumatic up-regulation of cutaneous cytokines remain unclear.

Animal studies demonstrate increased expression of neurokinin 1 (NK1) receptors in keratinocytes in the region of the limb, which was immobilized after the fracture. NK1 receptor stimulation leads to increased levels of inflammasomes and substance P (95). Both inflammasomes, which are multiprotein complexes, and substance P activate protease caspase-1 (96), which is responsible for the processing and activation of pro-inflammatory cytokines IL-1β, IL-18, and IL-33 (97). This results in increased cytokines leading to nociceptive sensitization and the development of CRPS. Keratinocytes in the immobilized limb express the increased transcription of NALP1, IL-1β, and caspase-1. Intraplanar injection of either IL-1β or IL-18 induced prolonged mechanical allodynia in a dose-dependent manner (69). Administration of a selective NK1 receptor antagonist (LY303870) partially reversed nociceptive and vascular changes observed with CRPS (92).

Clinical and preclinical evidence suggests that peripherally generated cytokines play a role in supporting CRPS I, particularly during its acute phases (98). Notably, there is compelling evidence highlighting the involvement of the IL-1 family of cytokines, with a focus on IL-1β, in modulating nociceptive information (99). IL-1β can exert its effects both directly on neurons and indirectly as an intermediate inflammatory mediator, contributing to the upregulation of nerve growth factor (NGF) (100) and other cytokines. Dysregulated activation of the NALP1 inflammasome in keratinocytes, triggered by fractures, leads to the abnormal release of IL-1β and IL-18. Additionally, an indirect mechanism via nerve growth factor is implicated. These substances collectively contribute to the nociceptive sensitization observed in the rat fracture model of CRPS I. While this inflammasome activity may not account for all manifestations of CRPS, such as limb warmth and edema, regulating inflammasomes and the associated signaling pathways offers promising avenues for innovative therapeutic approaches to address CRPS.

The release of these inflammatory mediators is associated with both the initial injury and the subsequent damage to cutaneous small nerves (CRPS Type I) and possibly to major nerves (CRPS Type II) (33). This is supported by evidence of a reduction in primary afferent C-type and Aδ-type fiber density in the CRPS-affected limb compared to the unaffected limb (101, 102). Consequently, there is an increase in aberrant fibers of unknown origin, leading to an exaggerated sensation of pain (101). A rat model illustrates the causal relationship between the exaggerated and aberrant neuronal triggers and the reduction in nerve fiber density resulting from the initial neuronal injury (103). The combined injury to both tissues and neurons contributes to the development of protective reflexes, giving rise to exaggerated inflammation and heightened responsiveness to pain. This phenomenon is a result of peripheral sensitization, a characteristic of CRPS that develops because of local tissue injury.

CRPS is further characterized by an increase in the proinflammatory cytokines TNF-α and MIP-1β (macrophage inflammatory protein-1 β), as well as a decrease in the anti-inflammatory cytokine IL-1RA (104). The interaction between cutaneous nerves and mast cells may contribute to the development of CRPS, and the loss of dermal nerve fibers could potentially attenuate chemotactic signals (105). Anti-inflammatory T-cell shifts, such as the decrease in Th17 regulated by CD39^+^ Tregs, may also serve as a mechanism for CRPS (106). As a result, targeting the processes and molecules involved in inflammation and autoimmunity could potentially lead to more effective treatments for CRPS.

### Oxidative stress

It has also been postulated that free radical generation by the mitochondrial respiratory chain is involved in the pathophysiology of CRPS I (107). Significant elevations in malondialdehyde, lactic dehydrogenase, and various antioxidants (peroxidase, superoxide dismutase, uric acid) in the serum and particularly the saliva of CRPS Type I patients were observed when compared to healthy individuals (91). Heightened levels of malondialdehyde were detected in the hind paw muscles in a rat model of CRPS (108). The pain hypersensitivity in these animals can be alleviated through the administration of free radical scavengers and antioxidant therapy (109). Despite extensive trials and convincing evidence of the association of reactive oxygen species and oxidative stress with CRPS, it has been challenging to ascertain whether it serves as a cause or consequence of the condition.

Oxidative stress arises from an imbalance between the production of reactive oxygen species (ROS) and the defense provided by antioxidants. Oxygen derivatives, specifically superoxide anion (O2–•), hydroxyl radical (OH–), and hydrogen peroxide (H2O2), along with reactive nitrogen species like nitric oxide and peroxynitrite, constitute the most significant free radicals. In regular physiological conditions, a variety of inherent biological mechanisms exist to counteract alterations in redox equilibrium, which include superoxide dismutase (SOD), catalase, and other antioxidant enzymes. The activation of antioxidant systems restrains free radical generation and terminates oxidative stress. However, when the production of ROS exceeds a certain pathological threshold over a specific duration, it can overwhelm the antioxidant defense and impair cellular functions. Since mitochondria serve as the primary origin of ROS, it is logical to conjecture that mitochondrial dysfunction associated with oxidative stress may contribute to the development of CRPS. Dysfunction in the mitochondria has also been recognized as a significant factor in the pathogenesis of degenerative disorders such as Alzheimer's disease, aging, diabetes, and ischemia-reperfusion injury (110). Mitochondria obtained from CRPS-I muscle tissue displayed reduced mitochondrial ATP production and substrate oxidation rates resulting in reduced mitochondrial energy production in comparison to control muscle tissue, suggesting that ROS-induced damage in muscle tissue mitochondria (107). ROS evoked damage to mitochondrial proteins and reduced manganese sodium dismutase (Mn SOD) levels and increased venous oxygen saturation levels have also been demonstrated in patients with chronic CRPS I, suggesting impaired oxygen diffusion and mitochondrial dysfunction associated with CRPS I (50).

Suboptimal nuclear factor erythroid 2-related factor 2 (Nrf2) activity may be implicated in a specific subset of patients with CRPS (111). Nrf2 is a transcription factor with a basic leucine zipper motif. It forms a heterodimeric complex with the antioxidant-responsive element (ARE) in the promoter regions of various cytoprotective genes. Nrf2 plays a pivotal role in the up-regulation of antioxidant, anti-inflammatory, and cell type-specific genes that are essential for the defense system.

### Autoimmunity

An important recent finding is that autoimmunity has a significant role in the development of CRPS. This is supported by compelling evidence such as the detection of immunoglobulin G (IgG) autoantibodies targeting surface antigens on autonomic neurons in the bloodstream of 70% of CRPS patients (112–114). These IgG antibodies potentially possess β2-adrenergic and muscarinic-2 receptor functionality. There exists activating antibodies against α-1a adrenoceptor in CRPS (113). It was shown that IgG autoantibodies from patients with severe, persistent CRPS, on transfer to hind paw-injured mice, elicit important features of the clinical condition and profound glial activation in pain-related brain regions (115, 116). Blockade of the proinflammatory cytokine interleukin-1 (IL-1) both prevents and reverses these changes. These findings suggest that antibody-mediated autoimmunity contributes to the development of severe CRPS after injury and that blockade of IL-1 actions may be an attractive therapeutic prospect.

IgM has been shown to contribute to nociception sensitization in addition to IgG. In a tibial fracture CRPS mouse model, mice that lacked B cells and IgM had attenuated nociceptive and inflammatory changes at 3 weeks post-fracture (117). Injecting IgM antibodies from mice with acute tibial fractures into CRPS mouse models, lacking B cells and IgM, produced pronociceptive effects (118). This lends further support to the hypothesis that autoimmunity is a likely contributor to the progression of CRPS. Moreover, this hypothesis is reinforced by the observation that patients who underwent immunoglobulin treatment experienced a notable decrease in pain symptoms compared to those who received a placebo (119). Thus, these antibodies have the potential to enhance the inflammatory response and nociception that is characteristic of CRPS.

Overactive immune reaction in response to inflammation leads to tissue damage in the acute phase of CRPS. Though the immune response is a normal physiological reaction to tissue damage, neuroimmune interactions and subsequent neuroinflammation tend to persist instead of diminishing in patients with CRPS. Both the innate and adaptive immune systems play a key role in the immune dysregulation observed in CRPS. With the innate immune system, activated keratinocytes, mast cells, and glial cells release proinflammatory cytokines, that can be detected in increased levels in blister fluid, serum, plasma, or cerebrospinal fluid of CRPS patients (88). These cytokines are associated with the activation and sensitization of peripheral nociceptors, leading to hyperalgesia and pain. Furthermore, the elevated levels of monocytes and their resident tissue macrophages in CRPS patients may serve as important innate cellular components (120). The involvement of the adaptive immune system is demonstrated by altered T-cell activity and a higher prevalence of autoantibodies found in CRPS patients (121).

CRPS could be regarded as an autoantibody-mediated autoimmune syndrome with a localized course (122). In autoimmune diseases, the innate immune system triggers an immune response by the adaptive immune system against its tissues. A significantly higher proportion of CRPS patients had positive antinuclear antibody test results compared to healthy blood bank donors (121). Additionally, CRPS patients showed the presence of IgG autoantibodies against surface antigens on autonomic neurons, which were absent in healthy controls. Topical application of serum-derived IgG obtained from patients with CRPS leads to the manifestation of mechanical allodynia and an elevation in tissue substance P (115). It is proposed that the immune response observed in individuals with CRPS generates autoantibodies targeting autonomic or sensory nerves, thereby contributing to the development of allodynia or heightened sensitivity within the affected area.

### Complement activation

Complements may play a critical role in the pathogenesis of CRPS (123). The complement cascade serves as a crucial element of the innate immune system and inflammation. In a meta-analysis encompassing 20 microarray studies investigating alterations in gene expression across diverse chronic pain models in rodents, complement emerged as among the most frequently and significantly regulated categories of genes, demonstrating upregulation after the induction of both neuropathic and inflammatory pain (124). It was further postulated in a mice model of CRPS that IgM antibodies bind to neoantigens in the fractured limb skin and corresponding spinal cord to activate C5a complement signaling in macrophages and microglia, evoking proinflammatory cytokine expression and contributing to nociceptive sensitization in the injured limb (125). Complement signaling assumes particular importance in directing neuronal responses to tissue injury, neurotrauma, and nerve lesions. It is increasingly recognized that complement orchestrates numerous host processes, notably those related to the functioning of the nervous system in both health and disease (126). Under normal physiological conditions, these processes include complement-dependent regulation of synaptic remodeling, axonal regrowth, neuronal damage, nociceptor sensitization, and pain. However, dysregulation of the complement cascade in various pathologies results in chronic inflammation, persistent pain, and neural dysfunction. There should exist checks and balances for the activation and inactivation of complements via the classical and alternative pathways. To prevent uncontrolled inflammation, autoimmunity, and the destruction of healthy tissues, rapid and extensive activation of the complement system in response to foreign invaders and injury necessitates equally potent and coordinated mechanisms to limit its activity. Nevertheless, the aberrant activation of the complement cascade has been implicated in fostering the progression of conditions marked by chronic pain, including complex regional pain syndrome and neuropathic pain (104, 114). The sustained or dysregulated signaling of complement factors observed in chronic pain suggests a plausible involvement of complement in the maladaptive mechanisms underlying CRPS.

### Neuroinflammation and neuroplasticity

Different from local tissue or systemic inflammation, neuroinflammation is localized inflammation in the PNS and CNS. It is characterized by the activation of microglia in the CNS or macrophages in the PNS. Microglia play a crucial role in coordinating the immune response within the CNS (59). Neuroinflammation can be triggered by various types of traumas or heightened neuronal activity in primary afferent nerve fibers or higher-order neurons. A positron emission tomography study demonstrated increased microglial activity in several brain regions of CRPS patients (127).

Neurogenic inflammation is a specific phenomenon in which nociceptive C-fibers that have been stimulated release neuropeptides, including substance P and CGRP. In patients with CRPS, the levels of CGRP and substance P in the blood were found to be higher compared to healthy individuals (61). The increase in neuropeptides can potentially account for some of the observed symptoms of CRPS, as these neuropeptides are known to induce vasodilation, protein extravasation, and sweating, and exert an influence on local immune cells and neural structures. It is noteworthy that this can contribute to the persistence of pain by facilitating central sensitization. In CRPS patients, the HPA axis may be impaired because of neuroinflammation. Decreased levels of cortisol and disrupted diurnal cortisol rhythms were observed in CRPS patients experiencing frequent pain attacks, indicating abnormal functioning of the HPA axis (128). As the HPA axis operates through a self-regulating negative feedback system, reduced cortisol levels may signify decreased activity or impaired feedback sensitivity of the HPA axis.

As the disease advances, there are persistent morphological alterations in the PNS. The examination of peripheral nerves utilizing transmission electron microscopy in a patient with CRPS revealed the differential degeneration of Aɑ fibers (motor/proprioception) and C fibers (nociception) while sparing Aδ fibers (nociception) (65). Degeneration of Aα fibers may lead to an imbalance in nerve signaling, inappropriately triggering the smaller healthy Aδ fibers, which transmit pain and temperature. Thus, peripheral nerve degeneration may play a key role in CRPS. This same process may induce changes in nociceptive processing within the CNS and heighten the excitability of secondary central nociceptive neurons in the spinal cord. Both central and peripheral sensitization processes are facilitated by the release of neuropeptides like substance P, bradykinin, and glutamate by peripheral nerves (129). These neurotransmitters sensitize and enhance the activity of local peripheral and secondary central nociceptive neurons, ultimately resulting in increased pain sensitivity to noxious stimuli (hyperalgesia) and pain in response to non-noxious stimuli (allodynia) (130). Individuals with CRPS exhibit a notably higher windup response to repeated stimulation of the affected limb when compared to the contralateral limb or other limbs (131, 132). Glutamate and substance-P are secreted in reaction to a neuroinflammatory reaction, causing a reduction in the threshold for reaction to mechanical stimuli (133). Consequently, this leads to heightened sensitivity in the peripheral nerve and intensified synaptic nociceptive signaling in the dorsal horn (134).

Neuroplasticity takes place in patients with CRPS. A decline in the somatosensory cortex was observed when comparing the limb affected by CRPS to the unaffected limb (135). Moreover, the somatosensory representation of the affected limb undergoes a reduction in size and experiences distortion (49). On occasion, the cortical area may undergo a shift. CRPS was once regarded as an aberration in the neuroplasticity of cortical function. Greater representation of an injured organ in the brain's cortex was linked to a higher incidence and severity of CRPS. This is exemplified by the observation that open wounds in the upper extremity carried a higher risk of CRPS compared to other body parts (odds ratio 1.53, 95% confidence interval 1.25 to 1.88, *p* < 0.05), given the substantial portion occupied by upper extremities in our cortical sensory homunculus.

### Neuroimaging of CRPS

Imaging studies have also shown that patients with CRPS may have decreased gray matter volume in the dorsal insula, left orbitofrontal cortex, and cingulate cortex, and increased gray matter volume in the bilateral dorsal putamen and right hypothalamus (136). The duration of pain exhibited a connection with a reduction in gray matter within the left dorsolateral prefrontal cortex while the intensity of pain demonstrated a negative correlation with volume in both sides of the dorsolateral prefrontal cortex. Simultaneously, the intensity of pain demonstrated a positive correlation with the volume in the left posterior hippocampus and left amygdala (137). A direct correlation has been established between the degree of reorganization and the intensity of pain as well as the extent of hyperalgesia reported by the patient. Interestingly, these alterations revert to their normal state upon successful treatment of CRPS (138). Pain may be triggered in certain individuals with CRPS by the mere act of contemplating the motion of the affected region (139). There exists an aberration in the primary motor cortex, supplementary motor cortices, posterior parietal cortices, and basal ganglia (140), which could plausibly explain certain manifestations such as dystonia and decreased range of motion observed in patients with CRPS. Moreover, CRPS has been discovered to hinder the capacity to perceive the physical movements performed by individuals, due to its impact on brain regions responsible for pain perception and motor regulation. Consequently, this results in the CRPS patient perceiving the actions of others as disagreeable or distressing (141). In summary, there are dysfunctional changes observed in the primary motor cortex, supplementary motor cortices, posterior parietal cortices, and basal ganglia, which may potentially contribute to and explain certain manifestations observed in patients with CRPS (140).

Positron emission tomography (PET) of translocator protein-18 kDa (TSPO) is a noninvasive technique utilized to monitor the activation of innate immune cells (142). The application of whole-body TSPO-PET, a highly adaptable technique, facilitates the visualization and measurement of peripheral and central myeloid lineage activation throughout the progression of the disease, offering insights into early and late disease stages (127). This method enables the longitudinal tracking of myeloid cell activation in both peripheral and central regions during the transition from acute to chronic pain. PET also allows for the observation of the spatiotemporal patterns of the innate immune response to injury, highlighting the early and sustained involvement of peripheral myeloid cells at the injury site over 2 days to 7 weeks, as well as the early and temporary activation of CNS microglia in regions distant from the injury site at 7- and 21-days post-injury. Understanding the role of myeloid cells in the shift from acute to chronic pain and the development of CRPS is crucial for the development of targeted treatments. The elevated expression of TSPO on activated myeloid cells following injury signifies the presence of inflammation, establishing it as a valuable biomarker for innate immune activation in various diseases. Utilizing a TSPO-PET tracer allows for the quantification of neuroinflammation (143). Previous investigations have demonstrated the involvement of microglia and astrocytes in the acute (0–4 weeks) vs. chronic (5–20 weeks) phases of CRPS, respectively (144).

The contribution of microglial activation to the development of CRPS was demonstrated in a mouse model that mirrors clinical conditions (145). Both male and female animals displayed activation of spinal cord microglia, indicated by increased levels of Iba1. However, the activation was less pronounced and delayed in female animals, which correlates with the findings of a study by Sorge et al. Who found sex specific role of TLR4 in inflammatory and neuropathic pain (146). The expression of a newly identified marker specific to microglia, TMEM119, was found in two distinct populations within the spinal cord parenchyma following peripheral injury: TMEM119+ microglia and TMEM119- infiltrating myeloid lineage cells, consisting of Ly6G+ neutrophils and Ly6G- macrophages/monocytes. Inflammatory mediators released in the CNS after injury sensitize neurons; spinal cord TMEM119+ microglia were found to be the origin of cytokines IL6 and IL1β following peripheral injury (145). Thus, targeting microglia to suppress its cytokine release may be an effective approach for alleviating pain (147).

### Genetic pathophysiology

Studies have indicated that there exists a close relationship between complex regional pain syndrome (CRPS) and the targeting of inflammatory genes (123). Prior investigations discovered associations between CRPS patients and HLA-A29.1, MMP9, ANPEP, HDC, G-CSF3R, and STAT3 (37); however, they neglected to carry out subsequent analyses of protein-protein interaction networks and gene set enrichment analysis (GSEA).

An extensive analysis of substantial quantities of data extracted from the GEO database ascertained the identification of pivotal genes and principal pathways that could potentially be employed in the development of novel clinical treatment strategies. The study further explored the examination and elucidation of the genetic foundations underlying the molecular mechanisms and pathogenesis of CRPS. The series matrix and corresponding platform information (GPL10558) were obtained from the NCBI website, which houses high-throughput gene expression data, chips, and microarrays under the accession number GSE47603 (123). In the CRPS group vs. the control, a total of thirty-seven differentially expressed genes (DEGs) were identified, with thirty-three being upregulated and four being downregulated. Upon conducting a molecular function (MF) analysis, it was discovered that the DEGs primarily play a role in peptide antigen binding, integrin binding, and actin filament binding. Further analysis through GO functional and KEGG enrichment methods demonstrated that the majority of the overlapping DEGs were mainly enriched in their inflammatory response (123). By studying the association between CRPS and the complement system, as well as identifying the top five hub genes (MMP9, PTGS2, CXCL8, OSM, TLN1), this study successfully constructed a protein-protein interaction (PPI) network and suggested that targeting excessive inflammation could offer new therapeutic approaches for CRPS.

## Concluding remarks

CRPS can be considered from various pathophysiological mechanisms. It exhibits inter-individual heterogeneity and even intra-individual variability over time (50). While the disease typically follows an inciting event such as surgery, nerve compression, fracture, trauma, ischemia, and sprain (27), CRPS can also arise spontaneously in the extremities or other areas of the body such as the head and neck. Neuroplasticity, autonomic dysfunction, autoimmunity, oxidative stress, and other neuronal mechanisms have been postulated as pivotal factors in the pathogenesis of CRPS (54). Both genetic predisposition and environmental stress contribute to the development of CRPS and alterations in the PNS and the CNS are identified in patients (34, 148). Inflammation and neuroimmune interactions play a critical role in the development of CRPS. Autoimmune mechanisms include IgG-mediated neuroinflammation and IgM mediated enhancement of nociception. This article presents an overview of the contributing factors in the development of CRPS and emphasizes the need for deeper mechanistic understanding at the cellular, molecular, genetic, transcriptomic, and environmental levels. Continued research into these components will help shed light on this enigmatic disease and develop novel therapeutic options for CRPS.

## Appendix 1

Research diagnostic criteria (the “Budapest Criteria”) for complex regional pain syndrome.

General definition of the syndrome: Complex regional pain syndrome describes an array of painful conditions that are characterized by a continuing (spontaneous and/or evoked) regional pain that is seemingly disproportionate in time or degree to the usual course of any known trauma or other lesion. The pain is regional (not in a specific nerve territory or dermatome) and usually has a distal predominance of abnormal sensory, motor, sudomotor, vasomotor, and/or trophic findings. The syndrome shows variable progression over time.

To make the clinical diagnosis, the following criteria must be met:

• Continuing pain, which is disproportionate to any inciting event.
• Must report at least one symptom in three of the four following categories:
  ○ sensory—reports of hyperesthesia and/or allodynia
  ○ vasomotor—reports of temperature asymmetry and/or skin color changes and/or skin color asymmetry
  ○ sudomotor/edema—reports of edema and/or sweating changes and/or sweating asymmetry
  ○ motor/trophic—reports of decreased range of motion and/or motor dysfunction (weakness, tremor, dystonia) and/or trophic changes (hair, nail, skin).
• Must display at least one sign at the time of evaluation in two or more of the following categories:
  ○ sensory—evidence of hyperalgesia (to pinprick) and/or allodynia (to light touch and/or temperature sensation and/or deep somatic pressure and/or joint movement)
  ○ vasomotor—evidence of temperature asymmetry (>1°C) and/or skin color changes and/or asymmetry
  ○ sudomotor/edema—evidence of edema and/or sweating changes and/or sweating asymmetry
  ○ motor/trophic—evidence of a decreased range of motion and/or motor dysfunction (weakness, tremor, dystonia) and/or trophic changes (hair, nail, skin)
  There is no other diagnosis that better explains the signs and symptoms.

## References

[1] Wen B Pan Y Cheng J Xu L Xu J. The role of neuroinflammation in complex regional pain syndrome: a comprehensive review. J Pain Res. (2023) 16:3061–73. 10.2147/JPR.S423733 37701560 PMC10493102

[2] Paré A. Of the cure of wounds of the nervous system. The collected works of Ambroise Pare [Preprint] (1968).

[3] The Classic: Gunshot Wounds and Other Injuries of Nerves: Clinical Orthopaedics and Related Research®. (n.d.). Available online at: https://journals.lww.com/clinorthop/abstract/1982/03000/the_classic__gunshot_wounds_and_other_injuries_of.2.aspx (Accessed March 24, 2024).

[4] Injuries of nerves and their consequences—Digital Collections—National Library of Medicine (n.d.). Available online at: https://collections.nlm.nih.gov/catalog/nlm: nlmuid-66230920R-bk (Accessed March 24, 2024).

[5] Iolascon G de Sire A Moretti A Gimigliano F. Complex regional pain syndrome (CRPS) type I: historical perspective and critical issues. Clin Cases Miner Bone Metab. (2015) 12(Suppl 1):4–10. 10.11138/ccmbm/2015.12.3s.004 27134625 PMC4832406

[6] Sudeck P. Uber die acute entzundliche knochenatrophie. Arch Clin Chir. (1900) 62:147–56.

[7] Evans JA. Reflex sympathetic dystrophy; report on 57 cases. Ann Intern Med. (1947) 26(3):417–26. 10.7326/0003-4819-26-3-417 20288177

[8] Foisie PS. Traumatic arterial vasospasm. N Engl J Med. (1947) 237(9):295–302. 10.1056/NEJM194708282370901 20259948

[9] van der Laan L Goris RJA. Reflex sympathetic dystrophy. Hand Clin. (1997) 13(3):373–85. 10.1016/S0749-0712(21)00099-8 9279543

[10] The Management of Pain with Special Emphasis on the Use of A…: Anesthesia & Analgesia (n.d.). Available online at: https://journals.lww.com/anesthesia-analgesia/citation/1954/09001/the_management_of_pain_with_special_emphasis_on.1.aspx (Accessed March 24, 2024).

[11] Harvey AM. Classification of chronic pain—descriptions of chronic pain syndromes and definitions of pain terms. Clin J Pain. (1995) 11(2):163. 10.1097/00002508-199506000-00024

[12] Harden RN Bruehl S Stanton-Hicks M Wilson PR. Proposed new diagnostic criteria for complex regional pain syndrome. Pain Med. (2007) 8(4):326–31. 10.1111/j.1526-4637.2006.00169.x 17610454

[13] Kessler A Yoo M Calisoff R. Complex regional pain syndrome: an updated comprehensive review. NeuroRehabilitation. (2020) 47(3):253–64. 10.3233/NRE-208001 32986618

[14] Atkins RM Duckworth T Kanis JA. Algodystrophy following Colles’ fracture. J Hand Surg. (1989) 14(2):161–4. 10.1016/0266-7681_89_90118-6 2746114

[15] Veldman PH Reynen HM Arntz IE Goris RJ. Signs and symptoms of reflex sympathetic dystrophy: prospective study of 829 patients. Lancet. (1993) 342(8878):1012–6. 10.1016/0140-6736(93)92877-v 8105263

[16] Todorova J Dantchev N Petrova G. Complex regional pain syndrome acceptance and the alternative denominations in the medical literature. Med Princ Pract. (2013) 22(3):295–300. 10.1159/000343905 23171669 PMC5586735

[17] Bruehl S. Complex regional pain syndrome. BMJ (Clin Res Ed). (2015) 351:h2730. 10.1136/bmj.h2730 26224572

[18] van Rijn MA Marinus J Putter H Bosselaar SR Moseley GL van Hilten JJ. Spreading of complex regional pain syndrome: not a random process. J Neural Transm. (2011) 118(9):1301–9. 10.1007/s00702-011-0601-1 21331457 PMC3162139

[19] Maleki J LeBel AA Bennett GJ Schwartzman RJ. Patterns of spread in complex regional pain syndrome, type I (reflex sympathetic dystrophy). Pain. (2000) 88(3):259–66. 10.1016/S0304-3959(00)00332-8 11068113

[20] Arden RL Bahu SJ Zuazu MA Berguer R. Reflex sympathetic dystrophy of the face: current treatment recommendations. Laryngoscope. (1998) 108(3):437–42. https://doi.org/10.1097/00005537-199803000-00023 9504621

[21] Heir GM Nasri-Heir C Thomas D Puchimada BP Khan J Eliav E Complex regional pain syndrome following trigeminal nerve injury: report of 2 cases. Oral Surg Oral Med Oral Pathol Oral Radiol. (2012) 114(6):733–9. https://doi.org/10.1016/j.oooo.2012.06.001 23102799

[22] Oaklander AL. Development of CRPS after shingles: it’s all about location. Pain. (2012) 153(12):2309–10. 10.1016/j.pain.2012.09.003 23059053

[23] Gellman H Keenan MAE Stone L Hardy SE Waters RL Stewart C. Reflex sympathetic dystrophy in brain-injured patients. Pain. (1992) 51(3):307–11. https://doi.org/10.1016/0304-3959(92)90214-V 1491858

[24] Griffith JF Guggenberger R. Peripheral nerve imaging. In: Hodler J Kubik-Huch RA von Schulthess GK, editors. Musculoskeletal Diseases 2021–2024: Diagnostic Imaging. Cham, CH: Springer (IDKD Springer Series) (2021). p. 259–68. 10.1007/978-3-030-71281-5_18

[25] Johnson S Cowell F Gillespie S Goebel A. Complex regional pain syndrome what is the outcome? - a systematic review of the course and impact of CRPS at 12 months from symptom onset and beyond. Eur J Pain. (2022) 26(6):1203–20. https://doi.org/10.1002/ejp.1953 35435302 PMC9324966

[26] Harden NR Bruehl S Perez RSGM Birklein F Marinus J Maihofner C Validation of proposed diagnostic criteria (the “Budapest criteria”) for complex regional pain syndrome. Pain. (2010) 150(2):268–74. https://doi.org/10.1016/j.pain.2010.04.030 20493633 PMC2914601

[27] Bruehl S Harden RN Galer BS Saltz S Bertram M Backonja M External validation of IASP diagnostic criteria for complex regional pain syndrome and proposed research diagnostic criteria. International association for the study of pain. Pain. (1999) 81(1–2):147–54. 10.1016/s0304-3959(99)00011-1 10353502

[28] Lloyd ECO Dempsey B Romero L. Complex regional pain syndrome. Am Fam Physician. (2021) 104(1):49–55. 34264598

[29] de Mos M de Bruijn AG Huygen FJ Dieleman JP Stricker BH Sturkenboom MC. The incidence of complex regional pain syndrome: a population-based study. Pain. (2007) 129(1–2):12–20. 10.1016/j.pain.2006.09.008 17084977

[30] Lewis JS Kashif M Maan A Ciampi de Andrade D Casey M Moon JY Global series: complex regional pain syndrome: abstracts from the international association for the study of pain complex regional pain syndrome SIG virtual symposia 2021. Pain Rep (Baltimore, Md). (2023) 8(1):e1056. 10.1097/PR9.0000000000001056 PMC9845011 36699996

[31] Bruehl S Chung OY. How common is complex regional pain syndrome-type I? Pain. (2007) 129(1–2):1–2. 10.1016/j.pain.2007.02.017 17379409

[32] Beerthuizen A Stronks DL Van't Spijker A Yaksh A Hanraets BM Klein J Demographic and medical parameters in the development of complex regional pain syndrome type 1 (CRPS1): prospective study on 596 patients with a fracture. Pain. (2012) 153(6):1187–92. 10.1016/j.pain.2012.01.026 22386473

[33] Bruehl S. An update on the pathophysiology of complex regional pain syndrome. Anesthesiology. (2010) 113(3):713–25. 10.1097/ALN.0b013e3181e3db38 20693883

[34] de Rooij AM de Mos M Sturkenboom MC Marinus J van den Maagdenberg AM van Hilten JJ. Familial occurrence of complex regional pain syndrome. Eur J Pain. (2009) 13(2):171–7. 10.1016/j.ejpain.2008.04.004 18514555

[35] Shaikh SS Goebel A Lee MC Nahorski MS Shenker N Pamela Y Evidence of a genetic background predisposing to complex regional pain syndrome type 1. J Med Genet. (2024) 61(2):163–70. 10.1136/jmg-2023-109236 37816627 PMC10850724

[36] Modarresi S Aref-Eshghi E Walton DM MacDermid JC. Does a familial subtype of complex regional pain syndrome exist? Results of a systematic review. Can J Pain. (2019) 3(1):157–66. 10.1080/24740527.2019.1637249 35005404 PMC8730611

[37] Jin EH Zhang E Ko Y Sim WS Moon DE Yoon KJ Genome-wide expression profiling of complex regional pain syndrome. PLoS One. (2013) 8(11):e79435. 10.1371/journal.pone.0079435 24244504 PMC3828360

[38] Pohóczky K Kun J Szentes N Aczél T Urbán P Gyenesei A Discovery of novel targets in a complex regional pain syndrome mouse model by transcriptomics: TNF and JAK-STAT pathways. Pharmacol Res. (2022) 182:106347. 10.1016/j.phrs.2022.106347 35820612

[39] Beerthuizen A van ‘t Spijker A Huygen FJ Klein J de Wit R. Is there an association between psychological factors and the complex regional pain syndrome type 1 (CRPS1) in adults? A systematic review. Pain. (2009) 145(1–2):52–9. 10.1016/j.pain.2009.05.003 19573987

[40] Wager J Brehmer H Hirschfeld G Zernikow B. Psychological distress and stressful life events in pediatric complex regional pain syndrome. Pain Res Manag. (2015) 20(4):189–94. 10.1155/2015/139329 26035287 PMC4532204

[41] Speck V Smith A Johnson B Lee C Garcia D White E Increased prevalence of posttraumatic stress disorder in CRPS. Eur J Pain. (2017) 21(3):466–73. 10.1002/ejp.940 27650922

[42] Bean DJ Johnson MH Kydd RR. Relationships between psychological factors, pain, and disability in complex regional pain syndrome and low back pain. Clin J Pain. (2014) 30(8):647–53. 10.1097/AJP.0000000000000007 24135903

[43] Beerthuizen A Arnold R Birklein F Bruehl S Maihöfner C Stanton-Hicks M The association between psychological factors and the development of complex regional pain syndrome type 1 (CRPS1)–a prospective multicenter study. Eur J Pain. (2011) 15(9):971–5. 10.1016/j.ejpain.2011.02.008 21459637

[44] Pons T Perez J Patel N Ramirez M Smith R Garcia L Potential risk factors for the onset of complex regional pain syndrome type 1: a systematic literature review. Anesthesiol Res Pract. (2015) 2015:956539. 10.1155/2015/956539 25688265 PMC4321092

[45] Pereira DE Costa N Silva M Santos P Oliveira S Lima F Patients with preexisting anxiety and mood disorders are more likely to develop complex regional pain syndrome after fractures. Clin Orthop Relat Res. (2024) 482(2):222–30. 10.1097/CORR.0000000000002957 38133494 PMC10776154

[46] Harden RN McCabe CS Goebel A Massey M Suvar T Grieve S Complex regional pain syndrome: practical diagnostic and treatment guidelines, 5th edition. Pain Med. (2022) 23(Suppl 1):S1–S53. 10.1093/pm/pnac046 35687369 PMC9186375

[47] Su YC Guo YH Hsieh PC Lin YC. A meta-analysis and meta-regression of frequency and risk factors for poststroke Complex regional pain syndrome. Medicina (Kaunas). (2021) 57(11). 10.3390/medicina57111232 PMC8622266 34833449

[48] Żyluk A Mosiejczuk H. A comparison of the accuracy of two sets of diagnostic criteria in the early detection of complex regional pain syndrome following surgical treatment of distal radial fractures. J Hand Surg Eur. (2013) 38(6):609–15. 10.1177/1753193412469142 23234766

[49] Marinus J Moseley GL Birklein F Baron R Maihöfner C Kingery WS Clinical features and pathophysiology of complex regional pain syndrome. Lancet Neurology. (2011) 10(7):637–48. 10.1016/S1474-4422(11)70106-5 21683929 PMC5511749

[50] Taha R Blaise GA. Update on the pathogenesis of complex regional pain syndrome: role of oxidative stress. Can J Anaesthesia. (2012) 59(9):875–81. 10.1007/s12630-012-9748-y 22798149

[51] Dey S Guthmiller KB Varacallo M. Complex Regional Pain Syndrome. In: StatPearls. Treasure Island, FL: StatPearls Publishing (2024). 28613470

[52] Parkitny L Middleton S Patel M Di Pietro F Cocco R Di Pietro L Inflammation in complex regional pain syndrome: a systematic review and meta-analysis. Neurology. (2013) 80(1):106–17. 10.1212/WNL.0b013e31827b1aa1 23267031 PMC3589200

[53] Guo T-Z Wei T Goffena A Xie Y Wang H Xu X Oxidative stress contributes to fracture/cast-induced inflammation and pain in a rat model of Complex regional pain syndrome. J Pain. (2018) 19(10):1147–56. 10.1016/j.jpain.2018.04.006 29715519 PMC6163064

[54] Baron R Janig W Harden RN Ferrante FM Stanton-Hicks M Merskey H National institutes of health workshop: reflex sympathetic dystrophy/complex regional pain syndromes–state-of-the-science. Anesth Analg. (2002) 95(6):1812–6. 10.1097/00000539-200212000-00064 12456464

[55] Shim H Lee CJ Kim W Oh SY Kim MK Yang YJ Complex regional pain syndrome: a narrative review for the practising clinician. Br J Anaesth. (2019) 123(2):e424–33. 10.1016/j.bja.2019.03.030 31056241 PMC6676230

[56] Fitzcharles MA Cohen SP Clauw DJ Littlejohn G Usui C Häuser W. Nociplastic pain: towards an understanding of prevalent pain conditions. Lancet. (2021) 397(10289):2098–110. 10.1016/s0140-6736(21)00392-5 34062144

[57] Raja SN Carr DB Cohen M Finnerup NB Flor H Gibson S The revised international association for the study of pain definition of pain: concepts, challenges, and compromises. Pain. (2020) 161(9):1976–82. 10.1097/j.pain.0000000000001939 32694387 PMC7680716

[58] Henderson J. Updated guidelines on complex regional pain syndrome in adults (✰). J Plast Reconstr Aesthet Surg. (2019) 72(1):1–3. 10.1016/j.bjps.2018.08.017 30268742

[59] Ji RR Nackley A Huh Y Terrando N Maixner W. Neuroinflammation and central sensitization in chronic and widespread pain. Anesthesiology. (2018) 129(2):343–66. 10.1097/aln.0000000000002130 29462012 PMC6051899

[60] Birklein F Schmelz M. Neuropeptides, neurogenic inflammation and complex regional pain syndrome (CRPS). Neurosci Lett. (2008) 437(3):199–202. 10.1016/j.neulet.2008.03.081 18423863

[61] Birklein F Drummond PD Li W Schlereth T Albrecht N Finch PM The important role of neuropeptides in complex regional pain syndrome. Neurology. (2001) 57(12):2179–84. 10.1212/wnl.57.12.2179 11756594

[62] Gay AM Béréni N Legré R. Type I complex regional pain syndrome. Chir Main. (2013) 32(5):269–80. 10.1016/j.main.2013.07.011 24094569

[63] de Rooij AM Perez J Patel N Ramirez M Smith R Garcia L Spontaneous onset of complex regional pain syndrome. Eur J Pain. (2010) 14(5):510–3. 10.1016/j.ejpain.2009.08.007 19793666

[64] Wang Y-C Chen Y-H Lee C-H Lin Y-C Huang L-L Yang Y-R Injury location and mechanism for complex regional pain syndrome: a nationwide population-based case-control study in Taiwan. Pain Pract. (2015) 15(6):548–53. 10.1111/papr.12211 24801059

[65] Yvon A Dufournet M Legré R Desmarais S De Cauwer H Broussolle E Selective fiber degeneration in the peripheral nerve of a patient with severe complex regional pain syndrome. Front Neurosci. (2018) 12:207. 10.3389/fnins.2018.00207 29670505 PMC5893835

[66] Krämer HH Maihöfner C Albrecht N Schlereth T Weiss T Birklein F TNF-α in CRPS and ‘normal’ trauma–significant differences between tissue and serum. Pain. (2011) 152(2):285–90. 10.1016/j.pain.2010.09.024 20947251

[67] Schwartzman RJ McLellan TL. Reflex sympathetic dystrophy. A review. Arch Neurol. (1987) 44(5):555–61. 10.1001/archneur.1987.00520170081028 3495254

[68] Terkelsen AJ Bach FW Jensen TS. Experimental forearm immobilization in humans induces cold and mechanical hyperalgesia. Anesthesiology. (2008) 109(2):297–307. 10.1097/ALN.0b013e31817f4c9d 18648239

[69] Li WW Guo TZ Wei T Goffena A Xie Y Wang H The NALP1 inflammasome controls cytokine production and nociception in a rat fracture model of complex regional pain syndrome. Pain. (2009) 147(1-3):277–86. 10.1016/j.pain.2009.09.032 19853379 PMC5515229

[70] Coderre TJ Bennett GJ Xie Y Wang H Li WW Guo TZ A hypothesis for the cause of complex regional pain syndrome-type I (reflex sympathetic dystrophy): pain due to deep-tissue microvascular pathology. Pain Med. (2010) 11(8):1224–38. 10.1111/j.1526-4637.2010.00911.x 20704671 PMC4467969

[71] Knudsen LF Terkelsen AJ Drummond PD Birklein F. Complex regional pain syndrome: a focus on the autonomic nervous system. Clin Auton Res. (2019) 29(4):457–67. 10.1007/s10286-019-00612-0 31104164

[72] Kortekaas MC Niehof SP Stolker RJ Huygen FJ. Pathophysiological mechanisms involved in vasomotor disturbances in Complex regional pain syndrome and implications for therapy: a review. Pain Pract. (2016) 16(7):905–14. 10.1111/papr.12403 26547635

[73] Groeneweg JG Niehof SP Stolker RJ Huygen FJ Xie Y Wang H Increased endothelin-1 and diminished nitric oxide levels in blister fluids of patients with intermediate cold type complex regional pain syndrome type 1. BMC Musculoskelet Disord. (2006) 7:91. 10.1186/1471-2474-7-91 17137491 PMC1693561

[74] Koban M Leis S Schultze-Mosgau S Birklein F Albrecht N Finch PM Tissue hypoxia in complex regional pain syndrome. Pain. (2003) 104(1-2):149–57. 10.1016/s0304-3959(02)00484-0 12855324

[75] Bussa M Guttilla D Lucia M Mascaro A Rinaldi S De Angelis L Adult complex regional pain syndrome type I: a narrative review. PM R. (2017) 9(7):707–19. 10.1016/j.pmrj.2016.11.006 27890578

[76] Misidou C Papagoras C. Complex regional pain syndrome: an update. Mediterr J Rheumatol. (2019) 30(1):16–25. 10.31138/mjr.30.1.16 32185338 PMC7045919

[77] Packham T Holly J. Complex regional pain syndrome: measurement matters: re: Galve-Villa M, Rittig-Rasmussen B, Mikkelsen LMS, Poulsen AG. Complex regional pain syndrome. Manual therapy 2016;26:e2–3. Man Ther. (2016) 26:e1. 10.1016/j.math.2016.07.012 27527554

[78] Birklein F Dimova V. Complex regional pain syndrome-up-to-date. Pain Rep. (2017) 2(6):e624. 10.1097/PR9.0000000000000624 29392238 PMC5741324

[79] Roberts WJ Smith A Johnson B Lee C Garcia D White E A hypothesis on the physiological basis for causalgia and related pains. Pain. (1986) 24(3):297–311. 10.1016/0304-3959(86)90116-8 3515292

[80] Cohen SP Patel N Ramirez M Perez J Brown A Martinez L Sympathetic blocks as a predictor for response to ketamine infusion in patients with Complex regional pain syndrome: a multicenter study. Pain Med. (2023) 24(3):316–24. 10.1093/pm/pnac153 36269190 PMC13396783

[81] Baron R Janig W Harden RN Ferrante FM Stanton-Hicks M Merskey H Relation between sympathetic vasoconstrictor activity and pain and hyperalgesia in complex regional pain syndromes: a case-control study. Lancet. (2002) 359(9318):1655–60. 10.1016/S0140-6736(02)08589-6 12020526

[82] Harden RN Duc TA Williams TR Coley D Cate JC Gracely RH. Norepinephrine and epinephrine levels in affected versus unaffected limbs in sympathetically maintained pain. Clin J Pain. (1994) 10(4):324–30. 10.1097/00002508-199412000-00014 7858364

[83] Kurvers H Daemen M Slaaf D Stassen F van den Wildenberg F Kitslaar P Partial peripheral neuropathy and denervation induced adrenoceptor supersensitivity. Functional studies in an experimental model. Acta Orthop Belg. (1998) 64(1):64–70. 9586253

[84] Finch PM Drummond ES Dawson LF Phillips JK Drummond PD. Upregulation of cutaneous α1 -adrenoceptors in complex regional pain syndrome type I. Pain Med. (2014) 15(11):1945–56. 10.1111/pme.12548 25220453

[85] Mailis-Gagnon A Bennett GJ. Abnormal contralateral pain responses from an intradermal injection of phenylephrine in a subset of patients with complex regional pain syndrome (CRPS). Pain. (2004) 111(3):378–84. 10.1016/j.pain.2004.07.019 15363882

[86] Cheng J Salmasi V You J Grille M Yang D Mascha EJ Outcomes of sympathetic blocks in the management of Complex regional pain syndrome: a retrospective cohort study. Anesthesiology. (2019) 131(4):883–93. 10.1097/aln.0000000000002899 31365367

[87] Xu J Yang J Lin P Rosenquist E Cheng J. Intravenous therapies for Complex regional pain syndrome: a systematic review. Anesth Analg. (2016) 122(3):843–56. 10.1213/ane.0000000000000999 26891396

[88] Huygen FJPM Ramdhani N Smith A Johnson B Lee C Garcia D Evidence for local inflammation in complex regional pain syndrome type 1. Mediat Inflamm. (2002) 11(1):47–51. 10.1080/09629350210307 PMC1781643 11930962

[89] Uçeyler N Ziegler D Patel N Ramirez M Perez J Brown A Differential expression patterns of cytokines in complex regional pain syndrome. Pain. (2007) 132(1–2):195–205. 10.1016/j.pain.2007.07.031 17890011

[90] Huygen FJPM Niehof SP Zijlstra FJ Stolker RJ Xie Y Wang H Successful treatment of CRPS 1 with anti-TNF. J Pain Symptom Manage. (2004) 27(2):101–3. 10.1016/j.jpainsymman.2003.12.006 15157033

[91] Eisenberg E Guo TZ Ziegler D Patel N Ramirez M Perez J Serum and salivary oxidative analysis in complex regional pain syndrome. Pain. (2008) 138(1):226–32. 10.1016/j.pain.2008.04.019 18539395

[92] Guo T-Z Wei T Goffena A Xie Y Wang H Xu X Substance P signaling contributes to the vascular and nociceptive abnormalities observed in a tibial fracture rat model of complex regional pain syndrome type I. Pain. (2004) 108(1–2):95–107. 10.1016/j.pain.2003.12.010 15109512

[93] Sabsovich I Guo TZ Wei T Goffena A Xie Y Wang H TNF signaling contributes to the development of nociceptive sensitization in a tibia fracture model of complex regional pain syndrome type I. Pain. (2008) 137(3):507–19. 10.1016/j.pain.2007.10.013 18035493 PMC2529181

[94] Li W-W Sabsovich I Wei T Goffena A Xie Y Wang H The role of enhanced cutaneous IL-1beta signaling in a rat tibia fracture model of complex regional pain syndrome. Pain. (2009) 144(3):303–13. 10.1016/j.pain.2009.04.033 19473768 PMC2743308

[95] Dallos A Kiss M Polyánka H Dobozy A Kemény L Husz S. Effects of the neuropeptides substance P, calcitonin gene-related peptide, vasoactive intestinal polypeptide and galanin on the production of nerve growth factor and inflammatory cytokines in cultured human keratinocytes. Neuropeptides. (2006) 40(4):251–63. 10.1016/j.npep.2006.06.002 16904178

[96] Mariathasan S Monack DM. Inflammasome adaptors and sensors: intracellular regulators of infection and inflammation. Nat Rev Immunol. (2007) 7(1):31–40. 10.1038/nri1997 17186029

[97] Dinarello CA. Interleukin-1 beta, interleukin-18, and the interleukin-1 beta converting enzyme. Ann N Y Acad Sci. (1998) 856:1–11. 10.1111/j.1749-6632.1998.tb08307.x 9917859

[98] Shi X Li W-W Wei T Sabsovich I Guo T-Z Kingery WS Neuropeptides contribute to peripheral nociceptive sensitization by regulating interleukin-1β production in keratinocytes. Anesth Analg. (2011) 113(1):175–83. 10.1213/ANE.0b013e31821a0258 21596883 PMC3123433

[99] Prasad Md A Chakravarthy Md K. Review of complex regional pain syndrome and the role of the neuroimmune axis. Mol Pain. (2021) 17:17448069211006616. 10.1177/17448069211006617 PMC8020088 33788654

[100] Martinon F Mayor A Tschopp J. The inflammasomes: guardians of the body. Annu Rev Immunol. (2009) 27:229–65. 10.1146/annurev.immunol.021908.132715 19302040

[101] Albrecht PJ Hines S Smith A Lee J Zhan M Hsieh ST Pathologic alterations of cutaneous innervation and vasculature in affected limbs from patients with complex regional pain syndrome. Pain. (2006) 120(3):244–66. 10.1016/j.pain.2005.10.035 16427199

[102] Oaklander AL Rissmiller JG Gelman LB Zheng L Chang Y Gott R. Evidence of focal small-fiber axonal degeneration in complex regional pain syndrome-I (reflex sympathetic dystrophy). Pain. (2006) 120(3):235–43. 10.1016/j.pain.2005.09.036 16427737

[103] Siegel SM Lee JW Oaklander AL. Needlestick distal nerve injury in rats models symptoms of complex regional pain syndrome. Anesth Analg. (2007) 105(6):1820–9. table of contents. 10.1213/01.ane.0000295234.21892.bc 18042888

[104] Lenz M Uçeyler N Frettloh J Höffken O Krumova EK Lissek S Local cytokine changes in complex regional pain syndrome type I (CRPS I) resolve after 6 months. Pain. (2013) 154(10):2142–9. 10.1016/j.pain.2013.06.039 23811041

[105] Morellini N Finch PM Goebel A Drummond PD Morellini N Plantaric K Dermal nerve fibre and mast cell density, and proximity of mast cells to nerve fibres in the skin of patients with complex regional pain syndrome. Pain. (2018) 159(10):2021–9. 10.1097/j.pain.0000000000001304 29905655

[106] Heyn J Azad SC Luchting B. Altered regulation of the T-cell system in patients with CRPS. Inflammation Res. (2019) 68(1):1–6. 10.1007/s00011-018-1182-3 30155690

[107] Tan EC Janssen AJ Roestenberg P van den Heuvel LP Goris RJ Rodenburg RJ. Mitochondrial dysfunction in muscle tissue of complex regional pain syndrome type I patients. Eur J Pain. (2011) 15(7):708–15. 10.1016/j.ejpain.2010.12.003 21262583

[108] Coderre TJ Xanthos DN Francis L Bennett GJ. Chronic post-ischemia pain (CPIP): a novel animal model of complex regional pain syndrome-type I (CRPS-I; reflex sympathetic dystrophy) produced by prolonged hindpaw ischemia and reperfusion in the rat. Pain. (2004) 112(1–2):94–105. 10.1016/j.pain.2004.08.001 15494189

[109] Zollinger PE Tuinebreijer WE Breederveld RS Kreis RW. Can vitamin C prevent complex regional pain syndrome in patients with wrist fractures? A randomized, controlled, multicenter dose-response study. J Bone Joint Surg Am. (2007) 89(7):1424–31. 10.2106/JBJS.F.01147 17606778

[110] Wallace DC. A mitochondrial paradigm of metabolic and degenerative diseases, aging, and cancer: a Dawn for evolutionary medicine. Annu Rev Genet. (2005) 39:359–407. 10.1146/annurev.genet.39.110304.095751 16285865 PMC2821041

[111] Baird L Dinkova-Kostova AT. The cytoprotective role of the Keap1-Nrf2 pathway. Arch Toxicol. (2011) 85(4):241–72. 10.1007/s00204-011-0674-5 21365312

[112] Kohr D Tschernatsch M Schmitz K Singh P Kaps M Schäfer KH Autoantibodies in complex regional pain syndrome bind to a differentiation-dependent neuronal surface autoantigen. Pain. (2009) 143(3):246–51. 10.1016/j.pain.2009.03.009 19375222

[113] Dubuis E Thompson V Leite MI Blaes F Maihöfner C Greensmith D Longstanding complex regional pain syndrome is associated with activating autoantibodies against alpha-1a adrenoceptors. Pain. (2014) 155(11):2408–17. 10.1016/j.pain.2014.09.022 25250722

[114] Birklein F Drummond PD Li W Schlereth T Albrecht N Finch PM Complex regional pain syndrome—phenotypic characteristics and potential biomarkers. Nat Rev Neurol. (2018) 14(5):272–84. 10.1038/nrneurol.2018.20 29545626 PMC6534418

[115] Tékus V Hajna Z Borbély É Markovics A Bagoly T Szolcsányi J A CRPS-IgG-transfer-trauma model reproducing inflammatory and positive sensory signs associated with complex regional pain syndrome. Pain. (2014) 155(2):299–308. 10.1016/j.pain.2013.10.011 24145209

[116] Helyes Z Pintér E Sándor K Elekes K Bánvölgyi A Keszthelyi D Transfer of complex regional pain syndrome to mice via human autoantibodies is mediated by interleukin-1-induced mechanisms. Proc Natl Acad Sci U S A. (2019) 116(26):13067–76. 10.1073/pnas.1820168116 31182576 PMC6600930

[117] Li WW Guo TZ Shi X Czirr E Stan T Sahbaie P Autoimmunity contributes to nociceptive sensitization in a mouse model of complex regional pain syndrome. Pain. (2014) 155(11):2377–89. 10.1016/j.pain.2014.09.007 25218828 PMC4252476

[118] Guo T-Z Wei T Li WW Li XQ Clark DJ Kingery WS Passive transfer autoimmunity in a mouse model of complex regional pain syndrome. Pain. (2017) 158(12):2410–21. 10.1097/j.pain.0000000000001046 28891866 PMC5680122

[119] Goebel A Baranowski A Maurer K Ghiai A McCabe C Ambler G A Randomised Placebo-Controlled Phase III Multicentre Trial: Low-Dose Intravenous Immunoglobulin Treatment for Long-Standing complex Regional Pain Syndrome (LIPS Trial). Southampton, UK: NIHR Journals Library (Efficacy and mechanism evaluation). (2017. 10.3310/eme04050 29144634

[120] Ritz BW Alexander GM Nogusa S Perreault MJ Peterlin BL Grothusen JR Elevated blood levels of inflammatory monocytes (CD14+ CD16 +) in patients with complex regional pain syndrome. Clin Exp Immunol. (2011) 164(1):108–17. 10.1111/j.1365-2249.2010.04308.x 21303362 PMC3074223

[121] Dirckx M Groeneweg G van Velzen M Huygen FJPM van Rijn M The prevalence of autoantibodies in complex regional pain syndrome type I. Mediat Inflamm. (2015) 2015:718201. 10.1155/2015/718201 PMC4337272 25741131

[122] Goebel A Blaes F. Complex regional pain syndrome, prototype of a novel kind of autoimmune disease. Autoimmun Rev. (2013) 12(6):682–6. 10.1016/j.autrev.2012.10.015 23219953

[123] Zhu H Guo T-Z Wei T Li WW Hou S Xie Y Identification of potential inflammation-related genes and key pathways associated with Complex regional pain syndrome. Biomolecules. (2023) 13(5). 10.3390/biom13050772 PMC10216790 37238642

[124] LaCroix-Fralish ML Austin JS Zheng FY Levitin DJ Mogil JS Sternberg WF. Patterns of pain: meta-analysis of microarray studies of pain. Pain. (2011) 152(8):1888–98. 10.1016/j.pain.2011.04.014 21561713

[125] Shi X Clark JD Kingery WS. C5a complement and cytokine signaling mediate the pronociceptive effects of complex regional pain syndrome patient IgM in fracture mice. Pain. (2021) 162(5):1400–15. 10.1097/j.pain.0000000000002150 33259455 PMC8049958

[126] Warwick CA Dutra SGB Valdearcos M Cheng T Yin W Ramalho T The complement cascade in the regulation of neuroinflammation, nociceptive sensitization, and pain. J Biol Chem. (2021) 297(3):101085. 10.1016/j.jbc.2021.101085 34411562 PMC8446806

[127] Jeon SY Kim JH Hong SB Lee SY. [11c]-(R)-PK11195 positron emission tomography in patients with complex regional pain syndrome: a pilot study. Medicine (Baltimore). (2017) 96(1):e5735. 10.1097/MD.0000000000005735 28072713 PMC5228673

[128] Park JY Ahn RS. Hypothalamic-pituitary-adrenal axis function in patients with complex regional pain syndrome type 1. Psychoneuroendocrinology. (2012) 37(9):1557–68. 10.1016/j.psyneuen.2012.02.016 22445364

[129] Wang H Wessendorf MW Anderson DJ. Bradykinin produces pain hypersensitivity by potentiating spinal cord glutamatergic synaptic transmission. J Neurosci. (2005) 25(35):7986–92. 10.1523/JNEUROSCI.2393-05.2005 16135755 PMC6725443

[130] Ji RR Woolf CJ. Neuronal plasticity and signal transduction in nociceptive neurons: implications for the initiation and maintenance of pathological pain. Neurobiol Dis. (2001) 8(1):1–10. 10.1006/nbdi.2000.0360 11162235

[131] Sieweke N Birklein F Riedl B Neundörfer B Handwerker HO. Patterns of hyperalgesia in complex regional pain syndrome. Pain. (1999) 80(1-2):171–7. 10.1016/s0304-3959(98)00200-0 10204729

[132] Eisenberg E Chistyakov AV Yudashkin M Kaplan B Hafner H Feinsod M Evidence for cortical hyperexcitability of the affected limb representation area in CRPS: a psychophysical and transcranial magnetic stimulation study. Pain. (2005) 113(1–2):99–105. 10.1016/j.pain.2004.09.030 15621369

[133] Schwartzman RJ Alexander GM Grothusen J. Pathophysiology of complex regional pain syndrome. Expert Rev Neurother. (2006) 6(5):669–81. 10.1586/14737175.6.5.669 16734515

[134] Woolf CJ. Central sensitization: implications for the diagnosis and treatment of pain. Pain. (2011) 152(3 Suppl):S2–15. 10.1016/j.pain.2010.09.030 20961685 PMC3268359

[135] Maihöfner C Handwerker HO Neundörfer B Birklein F. Patterns of cortical reorganization in complex regional pain syndrome. Neurology. (2003) 61(12):1707–15. 10.1212/01.wnl.0000098939.02752.8e 14694034

[136] Barad MJ Ueno T Younger J Chatterjee N Mackey S Complex regional pain syndrome is associated with structural abnormalities in pain-related regions of the human brain. J Pain. (2014) 15(2):197–203. 10.1016/j.jpain.2013.10.011 24212070 PMC4784981

[137] Ayoub LJ Honigman L Barnett AJ McAndrews MP Moayedi M. Mechanical pain sensitivity is associated with hippocampal structural integrity. Pain. (2024). 10.1097/j.pain.0000000000003221 39159941 PMC11331818

[138] Pleger B Dinse HR Ragert P Schwenkreis P Malin JP Tegenthoff M Sensorimotor retuning [corrected] in complex regional pain syndrome parallels pain reduction. Ann Neurol. (2005) 57(3):425–9. 10.1002/ana.20394 15732114

[139] Maihöfner C Handwerker HO Neundörfer B Birklein F Forster C Brain processing during mechanical hyperalgesia in complex regional pain syndrome: a functional MRI study. Pain. (2005) 114(1–2):93–103. 10.1016/j.pain.2004.12.001 15733635

[140] Azqueta-Gavaldon M Schulte-Göcking H Storz C Azad S Reiners A Borsook D Basal ganglia dysfunction in complex regional pain syndrome-a valid hypothesis? Eur J Pain. (2017) 21(3):415–24. 10.1002/ejp.975 27805769

[141] van Rijn MA Marinus J Putter H van Hilten JJ. Onset and progression of dystonia in complex regional pain syndrome. Pain. (2007) 130(3):287–93. 10.1016/j.pain.2007.03.027 17499924

[142] Cropper HC Johnson EM Haight ES Cordonnier SA Chaney AM Forman TE Longitudinal translocator protein-18 kDa-positron emission tomography imaging of peripheral and central myeloid cells in a mouse model of complex regional pain syndrome. Pain. (2019) 160(9):2136–48. 10.1097/j.pain.0000000000001607 31095093 PMC6527343

[143] Scholz J Woolf CJ. The neuropathic pain triad: neurons, immune cells and glia. Nat Neurosci. (2007) 10(11):1361–8. 10.1038/nn1992 17965656

[144] Sridharan S Lepelletier FX Trigg W Banister S Reekie T Kassiou M Comparative evaluation of three TSPO PET radiotracers in a LPS-induced model of mild neuroinflammation in rats. Mol Imaging Biol. (2017) 19(1):77–89. 10.1007/s11307-016-0984-3 27481358 PMC5209405

[145] Huck NA Siliezar-Doyle J Haight ES Ishida R Forman TE Wu S Temporal contribution of myeloid-lineage TLR4 to the transition to chronic pain: a focus on sex differences. J Neurosci. (2021) 41(19):4349–65. 10.1523/JNEUROSCI.1940-20.2021 33846230 PMC8143203

[146] Sorge RE LaCroix-Fralish ML Tuttle AH Sotocinal SG Austin JS Ritchie J Spinal cord toll-like receptor 4 mediates inflammatory and neuropathic hypersensitivity in male but not female mice. J Neurosci. (2011) 31(43):15450–4. 10.1523/JNEUROSCI.3859-11.2011 22031891 PMC3218430

[147] Greenhalgh AD Zarruk JG Healy LM Baskar Jesudasan SJ Jhelum P Salmon CK Peripherally derived macrophages modulate microglial function to reduce inflammation after CNS injury. PLoS Biol. (2018) 16(10):e2005264. 10.1371/journal.pbio.2005264 30332405 PMC6205650

[148] Taha R Blaise G. Is complex regional pain syndrome an inflammatory process? Theories and therapeutic implications. Can J Anaesthesia. (2007) 54(4):249–53. 10.1007/BF03022768 17400975

